# Supplementary material for: Systematic Investigation of the Effect of Lactobacillus acidophilus TW01 on Potential Prevention of Particulate Matter (PM)2.5-Induced Damage Using a Novel In Vitro Platform
Source: Foods. 2023 Sep 1;12(17):3278. doi: 10.3390/foods12173278 (PMC10486722; doi:10.3390/foods12173278)
Supplement: Supplementary file 1 [file foods-12-03278-s001.zip › foods-2548240-supplementary.pdf]

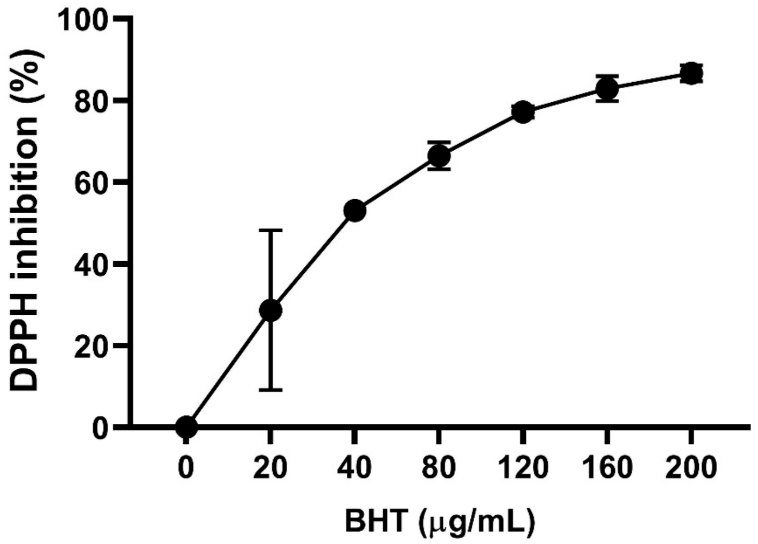

Figure S1. The butylated hydroxytoluene (BHT) standard curve for antioxidant assay (DPPH assay). The IC50 of BHT is 71.90 μg/mL. The data are presented as mean ± SD (n = 2).

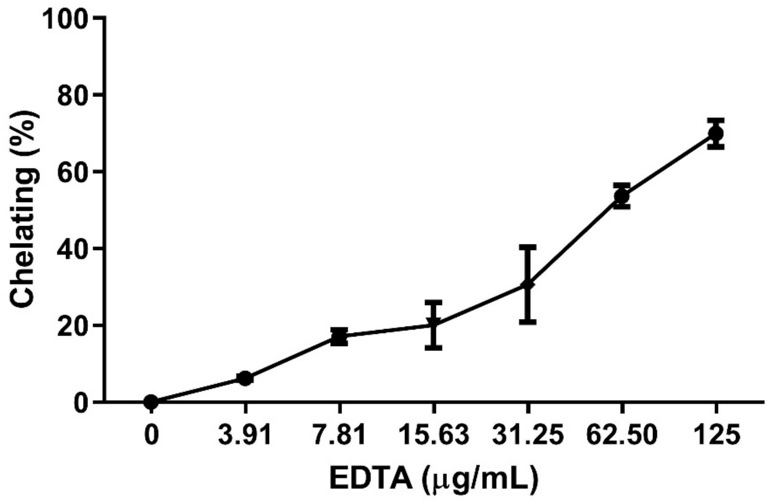

Figure S2. Ethylenediaminetetraacetic acid (EDTA) chelating rate in ferrozine ion chelating activity assay. The IC50 of EDTA is 58.24 μg/mL. The data are presented as mean ± SD (n = 2).
